# Health-Related Quality of Life and Its Influencing Factors for Elderly Patients With Hypertension: Evidence From Heilongjiang Province, China

**DOI:** 10.3389/fpubh.2021.654822

**Published:** 2021-03-16

**Authors:** Erwei Zheng, Jiao Xu, Juan Xu, Xueyun Zeng, Wan Jie Tan, Jinmei Li, Miaomiao Zhao, Bo Liu, Rui Liu, Mingjie Sui, Zhong Zhang, Yang Li, Hongbin Yang, Hongjuan Yu, Yongqing Wang, Qunhong Wu, Weidong Huang

**Affiliations:** ^1^The First Affiliated Hospital of Harbin Medical University, Harbin, China; ^2^School of Health Management, Harbin Medical University, Harbin, China; ^3^Duke-NUS Medical School, National University of Singapore, Singapore, Singapore; ^4^Heilongjiang Provincial Health Publicity Education and Information Center, Harbin, China; ^5^School of Public Health, Nantong University, Nantong, China; ^6^The Third Affiliated Hospital of Harbin Medical University, Harbin, China; ^7^Southern University of Science and Technology Hospital, Shenzhen, China; ^8^Heilongjiang University, Harbin, China

**Keywords:** hypertension, elderly patients, health-related quality of life, EQ-5D-3L, utility score

## Abstract

**Objective:** Hypertension is one of the most common public health issues worldwide. However, few existing studies examining health-related quality of life (HRQoL) were conducted on elderly patients with hypertension in China. Hence, this study aimed to assess the HRQoL of elderly patients with hypertension and its influencing factors using EuroQol five-dimensional-three-level (EQ-5D-3L) in China.

**Methods:** Data were obtained from the 6th National Health Service Survey in Heilongjiang province from June to July 2018, with a stratified multistage random cluster sampling method. All eligible participants were interviewed using a standardized questionnaire, which included the EQ-5D-3L, socio-demographics characteristics, clinical and lifestyle characteristics. The mean EQ-5D index scores for the different subgroups were evaluated using ANOVA. A Tobit regression model was also employed to analyze the potential factors influencing HRQoL.

**Results:** A total of 705 elderly patients with hypertension were included in this study. The mean EQ-5D utility score was 0.79 [standard deviation (SD) = 0.23]. The proportion of participants reporting pain/discomfort problems was the highest (57.0%), while problems in self-care was the lowest (17.2%). Influencing factors of HRQoL for elderly patients with hypertension included gender, age, income, education level, physical activity, health examination and coexisting diseases. Specifically, the female gender, being above 80 years old, having a lower education and/or higher income, and the presence of coexisting diseases were associated with lower utility index. In contrast, regular physical activity and medical examination had a positive impact on the HRQoL of elderly hypertension patients.

**Conclusion:** Overall, elderly patients with hypertension in China have a lower HRQoL than the general population. To improve the HRQoL of elderly patients with hypertension, it is imperative that better public health education is provided to enhance the knowledge of hypertension, encourage the adoption of healthy habits such as regular physical activity and medical examination, and improve the management of coexisting diseases. More care should also be directed to males with hypertension who are above 80 years old.

## Introduction

Hypertension is amongst the most common non-infectious chronic diseases. It has been identified as the leading risk factor for mortality, and is ranked third as a cause of disability-adjusted life years (DALYs) worldwide (1–3). It is estimated that up to 9.4 million pre-mature deaths and 92 million DALYs were attributable to hypertension each year (1), and is predicted that by the year 2025, there will be 1.56 billion people with hypertension (4). Likewise, hypertension as a public health challenge is similarly observed in China. A recent large population survey in China revealed that in 2019, ~245 million Chinese adults have hypertension (5). Moreover, hypertension tends to occur more frequently among older adults, where after the age of 69, the prevalence of hypertension rises to one in two individuals (6).

Health-related quality of life (HRQoL) is a concept commonly used in the subjective evaluation of a patient's health status, reflecting the patient's physical, psychological, social and emotional well-being (7, 8). As the HRQoL comprehensively examines the impact of the disease on the patient's life, as well as factors corresponding to their physical and mental health, a growing number of clinicians and policymakers are applying HRQoL in clinical treatment, drug research, preventive health care, health decision-making and health economic evaluation (9, 10). HRQoL can be measured using multi-attribute utility instruments (MAUI), which are divided into two categories: generic instruments and specific disease instruments (8, 11, 12). EuroQol five-dimension (EQ-5D) is one of the most commonly used generic instruments in the world. It has been translated into more than 170 languages and is used to measure the HRQoL of the general population and several patient populations (13). As a preference-based tool, EQ-5D can measure the health state utility (HSU) of the population to estimate the quality-adjusted life years (QALYs) (14). The three-level version of the EQ-5D (EQ-5D-3L) was introduced in 1990 (15). It has demonstrated validity and reliability (16–18), and has been widely used to measure the HRQoL of several medical conditions including hypertensive patients in China (16–25).

Previous studies have examined the relationship between HRQoL and hypertension in elder populations (26, 27). For example, a study in China reported that elderly patients with hypertension have low HRQoL (28). Another study in Vietnam found that advanced age and comorbidity were negatively associated with HRQoL (29). However, most of the studies conducted with Chinese elder population have measured HRQoL using generic instruments comprising of many items, such as the Medical Outcomes 36-Item Short Form Health Survey (SF-36) (30, 31). To date, few studies have evaluated the HRQoL of elderly patients with hypertension in China using well-validated MAUI, such as the EQ-5D.

Therefore, the current study aimed to: (1) estimate the HRQoL of elderly patients with self-reported diagnosis of hypertension, and (2) identify factors of the HRQoL that are associated with hypertension.

## Methods

### Study Design and Data Collection

Data was extracted from the 6th National Health Services Survey (NHSS) conducted in Heilongjiang, a province located in northeastern China with a population of 38.7 million and a middle-income economy in terms of its gross domestic product per capita (32).

NHSS is a cross-sectional household questionnaire survey conducted in China once every 5 years, and is overseen by the Center for Health Statistics Information. The 6th NHSS was conducted from June to July 2018.

In the 6th NHSS, a multi-stage stratified cluster random sampling method was adopted, involving 6,627 individuals from 3,000 households in five counties/districts, comprising of 25 towns/sub-districts and 50 villages/residential committees. Well-trained interviewers used standardized questionnaires installed on tablets to collect information. Each field site had a survey supervisor who revisited 5% of the participating households to validate the information that was collected (33).

The questionnaire included items on demographic (e.g., age, sex, and ethnicity) and socioeconomic (e.g., residency, marital status, educational attainment, employment, income, housing, and health insurance) data, clinical status (e.g., chronic conditions), and lifestyle (e.g., smoking, alcohol consumption, health examination, and physical activity) of the participants (34). Inclusion criteria for the participants were: (1) having answered “Yes” to the question “Have you been diagnosed with hypertension by a doctor?,” and (2) being 60 years old and above. This resulted in a final sample of 705 participants for the purpose of this study.

### Measurements

The EQ-5D-3L was used as a tool to measure the HRQoL of elderly patients with hypertension in the present study (15). The EQ-5D-3L contains a short health description system questionnaire (EQ-5D descriptive system) and a visual analog scale (EQ-VAS). EQ-VAS is used for respondent's own global rating of their overall health, on a scale from 0 (worst possible health) to 100 (best health possible). The description system of the EQ-5D-3L consists of five health dimensions: “Mobility,” “Self-care,” “Usual Activities,” “Pain/Discomfort,” and “Anxiety/Depression.” Each dimension has three response categories: “no problems,” “some problems,” and “extreme problems.” Thus, the EQ-5D-3L defines a total of 243 (3^5^) health states, with the best heath state indicated by the response “11111” and the worst health state indicated by the response “33333.” In the current study, EQ-5D-3L health states were (33) converted into a single healthy utility index score using a scoring algorithm that is based on the public preferences of the Chinese population (35).

### Statistical Analysis

We compared the utility scores of the FCs with those of the local general population norm which were available from a representative sample of the local population in Heilongjiang as part of the fourth National Health Services Survey, involving 15,875 individuals (from 5,530 households) in 13 cities and counties (33). To explore the factors associated with the HRQoL, health utility score of the patient were compared between the different socio-demographic groups (gender, age, area, level of education, marital status, medical insurance, annual household income, and employment status), which have been commonly used in studies on HRQoL of patients with hypertension (21, 26, 36). Consistent with other studies on HRQoL of patients with hypertension (20, 22, 37), comorbidity and lifestyle characteristics, such as smoking status, alcohol consumption, health examination, and physical activity, were also included as these are potential factors associated with HRQoL.

All data analyses were performed using STATA 15.0 software. Statistical significance was defined as *p* < 0.05. Descriptive statistics were calculated for the basic demographic variables of HRQoL. Mean and standard deviations (SD) were calculated for continuous variables, while frequencies and percentages were calculated for categorical variables. Student *t*-tests (for two groups) and one-way ANOVA tests (for multiple groups) were used to examine how EQ-5D utility scores may differ on different levels of each variable. All variables tested by the ANOVA-test were entered into the Tobit regression model. Previous studies recommend Tobit regression to deal with data of such a censored nature (38, 39), because they have theoretical advantages over the ordinary least squares estimator (40). In the present study, 34.1% of the respondents had the highest possible score of 1.0. As the EQ-5D utility data was censored, a Tobit regression model was hence employed to identify associated factors of HRQoL among elderly patients with hypertension.

### Results

The majority of the elderly patients with hypertension in the sample had the following characteristics: female (54%), rural residents (53.5%), primary school education level (42.35%), covered by medical insurance (95.7%), married (76.6%), and unemployed (47.9%). Respondents were less likely to smoke (74.8%), drink (79.7%), and take a health examination (51.9%). Furthermore, 57.2% of the respondents regularly participate in physical activities (Table 1).

**Table 1:** Characteristics of elderly patients with hypertension (*N* = 705).

**Characteristics**	***N* = 705**
**Gender (*****n*****, %)**	
Male	324 (46.0%)
Female	381 (54.0%)
Age group (years, mean ± SD)	67.9 ± 6.11
60-	477 (67.7%)
70-	189 (26.8%)
80-	39 (5.5%)
**Area (*****n*****, %)**	
Urban	328 (46.5%)
Rural	377 (53.5%)
**Education (*****n*****, %)**	
Illiterate	152 (21.6%)
Primary school	298 (42.3%)
Junior high school	153 (21.6%)
Senior high/technical school	81 (11.5%)
College and above	21 (3.0%)
**Medical insurance (*****n*****, %)**	
No	30 (4.3%)
Yes	675 (95.7%)
**Marital status (*****n*****, %)**	
Married	540 (76.6%)
Single	2 (0.3%)
Divorced	12 (1.7%)
Widowed	151 (21.4%)
**Employment status (*****n*****, %)**	
Employed	127 (18%)
Retired	240 (34.1%)
Unemployed	338 (47.9%)
**Smoking status (*****n*****, %)**	
No	527 (74.8%)
Yes	178 (25.2%)
**Drinking status (*****n*****, %)**	
No	562 (79.7%)
Yes	143 (20.3%)
**Physical activity (*****n*****, %)**	
No	302 (42.8%)
Yes	403 (57.2%)
**Health examination (*****n*****, %)**	
No	366 (51.9%)
Yes	339 (48.1%)
**Comorbidity (*****n*****, %)**	
No	295 (41.8%)
Yes	410 (58.2%)
**Income**	
Low	241 (34.2%)
Middle	216 (30.6%)
High	248 (35.2%)

The dimension of the EQ-5D-3L with the highest proportion of elderly patients reporting “no problem” was “self-care” at 82.8%, followed by “anxiety/depression” at 76.9%. As for the dimensions of “usual activities” and “mobility,” the proportion of respondents rating “no problem” were 71.1 and 60.2%, respectively. In contrast, the “Pain/Discomfort” dimension exhibited the lowest proportion of “no problem” responses (43.0%). Overall, a total of 241 participants (34.1%) reported “no problems” in any of the five dimensions (Table 2).

**Table 2:** Frequency of response in each dimension of the EQ-5D-3L (*N* = 705).

	**No problems**	**Moderate problems**	**Extreme problems**
Mobility (%)	60.2	36.5	3.3
Self-care (%)	82.8	13.2	4.0
Usual activities (%)	71.1	18.4	10.5
Pain/discomfort (%)	43.0	52.5	4.5
Anxiety/depression (%)	76.9	21.3	1.8

**Figure 1:**
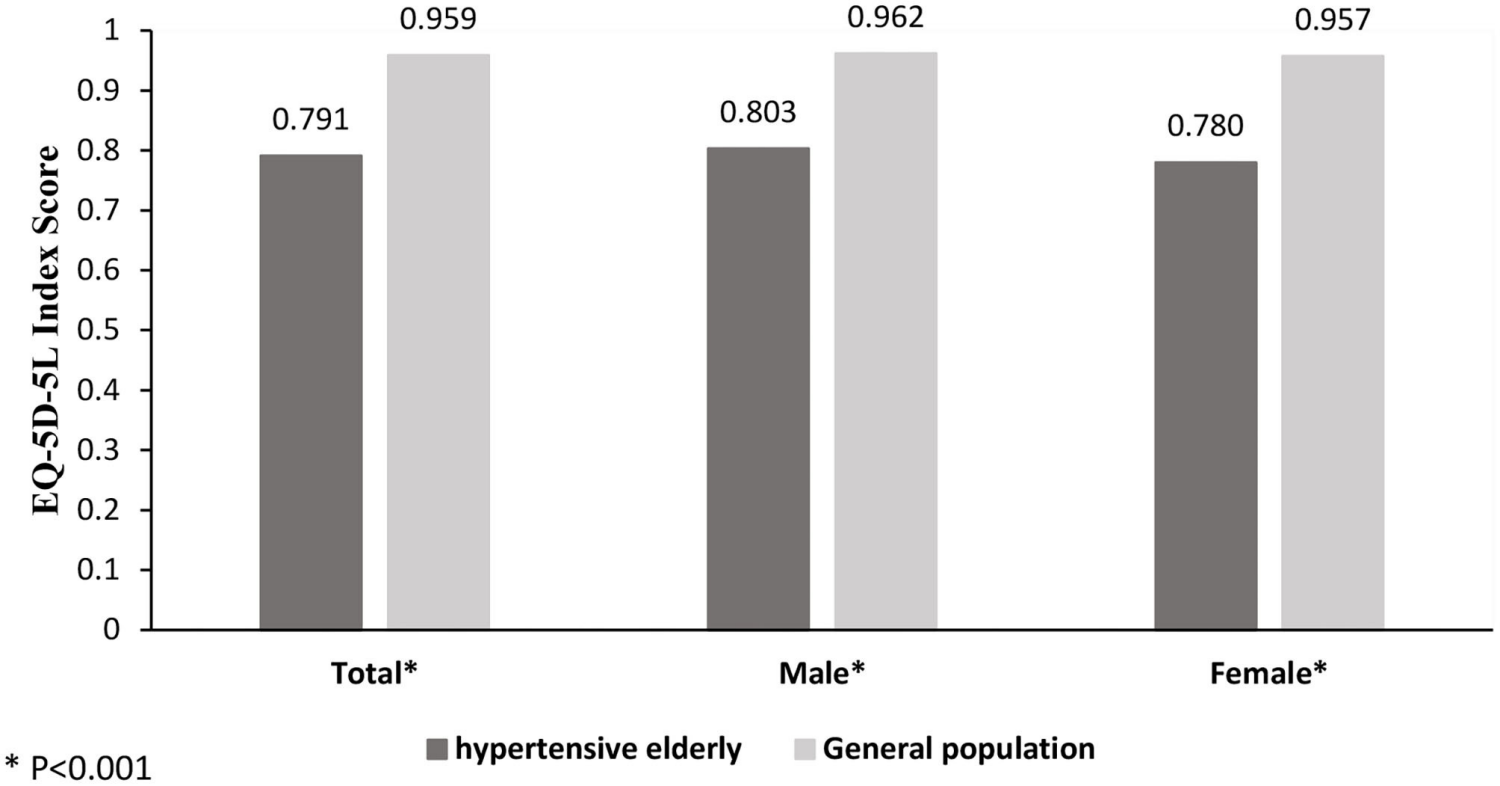
Health utility of elderly patients with hypertension compared to the general population.

Figure 1 depicts the comparison of the EQ-5D-3L utility scores between elderly patients with hypertension and the general population in Heilongjiang, China. Patients had a significantly lower utility score than the general population (0.79 vs. 0.96, *p* < 0.001). When classified by gender, the utility score of the patients were also significantly lower than those of their respective counterparts in the general population (*p* < 0.001).

Lower healthy utility index scores were found among respondents who were older (*p* < 0.001), lived in urban areas (*p* = 0.016), had a lower education level (*p* < 0.001), or were unemployed (*p* < 0.001). In contrast, higher utility scores were found in hypertensive respondents who drunk (*p* < 0.001), exercised regularly (*p* < 0.001), had no comorbidity (*p* < 0.001), and had a lower income level (*p* = 0.002). The index scores are described in Table 3.

**Table 3:** EQ-5D-3L health utility index scores on each characteristic of elderly patients with hypertension.

**Characteristics**	**Mean ± SD**	**Median (range)**	***P-*values**
Gender			0.183
Male	0.803 ± 0.220	0.869 (−0.149–1)	
Female	0.780 ± 0.234	0.862 (−0.149–1)	
Age group			<0.001^***^
60-	0.816 ± 0.209	0.869 (−0.149–1)	
70-	0.754 ± 0.243	0.783 (−0.149–1)	
80-	0.646 ± 0.293	0.610 (−0.149–1)	
Area			0.016^**^
Urban	0.771 ± 0.214	0.783 (−0.005–1)	
Rural	0.812 ± 0.241	0.869 (−0.149–1)	
Education			<0.001^***^
Illiterate	0.714 ± 0.262	0.770 (−0.149–1)	
Primary school	0.779 ± 0.221	0.862 (−0.149–1)	
Junior high school	0.845 ± 0.193	0.869 (−0.03–1)	
Senior high/ technical school	0.846 ± 0.216	0.869 (−0.149–1)	
College and above	0.897 ± 0.141	1.000 (0.45–1)	
Medical insurance			0.127
No	0.852 ± 0.197	0.869 (0.114–1)	
Yes	0.787 ± 0.229	0.869 (−0.149–1)	
Marital status			0.025^**^
Married	0.803 ± 0.222	0.869 (−0.149-1)	
Single	0.885 ± 0.163	0.885 (0.77–1)	
Divorced	0.741 ± 0.243	0.770 (−0.149–1)	
Widowed	0.818 ± 0.237	0.869 (0.22–1)	
Employment status			<0.001^***^
Employed	0.842 ± 0.181	0.869 (0.056–1)	
Retired	0.828 ± 0.226	0.869 (−0.149–1)	
Unemployed	0.744 ± 0.236	0.770 (−0.149–1)	
Smoking status			0.115
No	0.782 ± 0.239	0.869 (−0.149–1)	
Yes	0.814 ± 0.190	0.869 (0.22–1)	
Drinking status			<0.001^***^
No	0.773 ± 0.237	0.862 (−0.149–1)	
Yes	0.859 ± 0.168	0.869 (0.22–1)	
Physical activity			<0.001^***^
No	0.706 ± 0.271	0.770 (−0.149–1)	
Yes	0.854 ± 0.162	0.869 (0.22–1)	
Health examination			0.141
No	0.778 ± 0.245	0.869 (−0.149–1)	
Yes	0.803 ± 0.207	0.869 (−0.149–1)	
Comorbidity			<0.001^***^
No	0.874 ± 0.176	1.000 (−0.149–1)	
Yes	0.730 ± 0.241	0.770 (−0.149–1)	
Income			0.002^***^
Low	0.831 ± 0.196	0.870 (−0.005–1)	
Middle	0.767 ± 0.250	0.862 (−0.149–1)	
High	0.771 ± 0.231	0.857 (−0.149–1)	

* P < 0.1,

** P < 0.05,

*** *P < 0.01*.

Findings from the Tobit regression model further confirmed that gender, age, education level, physical activity, health examination, comorbidity, and income level were associated with the HRQoL of elderly patients with hypertension (Table 4).

**Table 4:** Factors influencing the EQ-5D-3L utility scores from the Tobit regression model.

**Variables**	**Coefficient**	**SE**	***P-*values**
**Gender**			
Male	Ref.		
Female	−0.059	0.024	0.017^**^
**Age group**			
60-	Ref.		
70-	−0.035	0.029	0.224
80-	−0.118	0.041	0.004^***^
**Area**			
Urban	Ref.		
Rural	−0.025	0.057	0.655
**Education**			
Illiterate	Ref.		
Primary school	0.062	0.025	0.015^**^
Junior high school	0.119	0.041	0.004^***^
Senior high/technical school	0.105	0.039	0.007^***^
College and above	0.146	0.073	0.046^**^
**Medical insurance**			
No	Ref.		
Yes	−0.035	0.049	0.476
**Marital status**			
Married	Ref.		
Single	0.293	0.189	0.122
Divorced	0.014	0.086	0.867
Widowed	−0.039	0.022	0.086
**Employment status**			
Employed	Ref.		
Retired	−0.072	0.053	0.176
Unemployed	−0.055	0.047	0.244
**Smoking status**			
No	Ref.		
Yes	0.027	0.032	0.397
**Drinking status**			
No	Ref.		
Yes	0.060	0.037	0.106
**Physical activity**			
No	Ref.		
Yes	0.149	0.044	0.001^***^
**Health examination**			
No	Ref.		
Yes	0.066	0.029	0.023^**^
**Comorbidity**			
No	Ref.		
Yes	−0.195	0.077	0.011^**^
**Income**			
Low	Ref.		
Middle	−0.088	0.042	0.037^**^
High	−0.060	0.029	0.037^**^

* P < 0.1,

** P < 0.05,

*** *P < 0.01*.

## Discussion

The present study quantified the utility scores of elderly patients with self-reported diagnosis of hypertension, and identified factors that influenced HRQoL using the EQ-5D-3L. To the best of our knowledge, this makes the present study the first of its kind in Heilongjiang province, China.

Findings showed that older adults with hypertension reported significantly more problems in each of the EQ-5D domains and have a lower health utility index than the local general population (33). This indicates that hypertension has an adverse effect on older mainland Chinese. Similar to previous studies, the present study found that the utility score was lower for respondents with hypertension than those without (19–21). Nonetheless, it is important to note that in previous studies, the mean utility score of the respondents with a diagnosis of hypertension were lower [i.e., 0.96 in Yao et al.'s (20), 0.92 in Zhang et al.'s (21), and 0.85 in Liang et al.'s (19) studies] than in the present study. Moreover, current respondents reported more problemsin all five dimensions of the EQ-5D-3L than reported in other studies (20, 21). This may be mainly due to differences in age. Participants in the current study were elderly patients aged 60 years and older, while previous studies included all adults over 18 years old. Moreover, the present study found that the HRQoL of those aged 60 years and above with hypertension gradually decreased with age. It is possible that hypertension is a chronic disease which progresses with age and increasingly affects health (41). The functions and immunity of the body gradually decline with age, and especially in the northeast of China where the winters are cold and long, the elderly people spend less time outside and have lesser physical activity (42). In addition, it is difficult for the elderly with high blood pressure to acquire knowledge on the management of hypertension, especially when information is largely obtained through the Internet in recent times (43).

Likewise, the HRQoL of elderly females with hypertension was also lower compared to their male counterparts in the present study. Similar gender differences in HRQOL of patients with hypertension have been found in Japan and South Korea populations (44, 45). This may be due to variances in social position and opportunities between males and females in different societies. In China, there are the gender inequities in areas such as socioeconomic status (20), education (21), and health (19). Hence, a low HRQoL among elderly females with hypertension would be expected.

The present study also found that patients with higher education levels demonstrated better HRQoL. This is consistent with the findings reported by Andrade et al. (46), Zhang et al. (21) and Saleem et al. (47). A possible explanation for this is that people with higher educational levels tend to have higher levels of health literacy, such as reducing salt intake, quitting smoking, restricting alcohol, and complying with medical advice, which are considered helpful for improving HRQoL (48).

Regular physical exercise is one of the important influencers of HRQoL in elderly people with hypertension, which is consistent with the conclusion of a previous study that utilized the SF-36 (49). Moreover, the HRQoL of patients with hypertension who underwent health examinations in the past year was significantly higher than that of patients who did not. These findings are consistent with the results in a previous study (20). Health management, such as regular physical exercise and medical examination, is beneficial in preventing and treating hypertension. For example, regular physical exercise could promote blood circulation and metabolism, reduce blood pressure, increase fat burning, and body shape maintenance (50). As for regular health examinations, it can help to detect chronic diseases including hypertension at an early stage, and help patients be mindful of their own health, improve their living habits, and pay attention to the prevention and treatment of hypertension and other comorbidities (20).

The present research also confirmed that elderly patients with hypertension and other comorbidities tended to have lower HRQoL, which is consistent with findings of previous studies (19, 20, 28, 51). Patients with hypertension are susceptible to a range of comorbidities, such as myocardial infarction, angina pectoris, stroke, and kidney failure, which is considered as one of main risk factors that reduce HRQoL (52). A previous study found that nearly 20% of the HRQoL scores could be caused by comorbidity, while only 2% of that are due to hypertension (53). It could be postulated that this variance of HRQoL is more serious in elderly patients with hypertension. Therefore, it is essential to consider commodity when evaluating HRQoL among elderly patients with hypertension, and prevent and treat comorbidity that may further decrease HRQoL.

We acknowledge that the current research has the following limitations. First, although the study sample is a regionally representation, findings from the present study may not be generalizable to other geographic areas in China. Second, as the present study utilized a cross-sectional design, it is difficult to ascertain the causal relationship between HRQoL and associated factors. Third, the cases of hypertension included in this study are restricted to those 60 years and above with a self-reported diagnosis of hypertension by a doctor, hence accuracy may be affected.

## Conclusions

Overall, elderly patients with hypertension in China have a lower HRQoL than the general population. To improve the HRQoL of elderly patients with hypertension, it is imperative that better public health education is provided to enhance the knowledge of hypertension, encourage the adoption of healthy habits such as regular physical activity and health examination, and improve the management of coexisting diseases. More care should also be directed to females with hypertension who are above 80 years old.

## Data Availability Statement

The data analyzed in this study is subject to the following licenses/restrictions: The data and code are available from the corresponding author upon reasonable request. Requests to access these datasets should be directed to weidong218@126.com.

## Author Contributions

YW, QW, and WH contributed to the conception and design of the study. EZ, JiX, JuX, JL, MZ, BL, RL, MS, ZZ, YL, HYa, and HYu conducted the data reduction and analyses. XZ and WH wrote the manuscript. YW, QW, WH, and WT reviewed the manuscript. All authors read and approved the manuscript before submission.

## Conflict of Interest

The authors declare that the research was conducted in the absence of any commercial or financial relationships that could be construed as a potential conflict of interest.
